# TCFL5 deficiency impairs the pachytene to diplotene transition during spermatogenesis in the mouse

**DOI:** 10.1038/s41598-022-15167-w

**Published:** 2022-06-29

**Authors:** Javier Galán-Martínez, Inés Berenguer, Mª del Carmen Maza, Konstantinos Stamatakis, Núria Gironès, Manuel Fresno

**Affiliations:** 1 Centro de Biología Molecular Severo Ochoa, Nicolás Cabrera 1, Cantoblanco, 28049 Madrid, Spain; 2 Consejo Superior de Investigaciones Científicas, Cantoblanco, Madrid, Spain; 3 Universidad Autónoma de Madrid, Cantoblanco, Madrid, Spain; 4 Instituto Sanitario de Investigación Princesa, Madrid, Spain

**Keywords:** Spermatogenesis, Meiosis

## Abstract

Spermatogenesis is a complex, multistep process during which spermatogonia give rise to spermatozoa. Transcription Factor Like 5 (TCFL5) is a transcription factor that has been described expressed during spermatogenesis. In order to decipher the role of TCFL5 during in vivo spermatogenesis, we generated two mouse models. Ubiquitous removal of TCFL5 generated by breeding TCFL5^fl/fl^ with SOX2-Cre mice resulted in sterile males being unable to produce spermatozoa due to a dramatic alteration of the testis architecture presenting meiosis arrest and lack of spermatids. SYCP3, SYCP1 and H1T expression analysis showed that TCFL5 deficiency causes alterations during pachytene/diplotene transition resulting in a meiotic arrest in a diplotene-like stage. Even more, TCFL5 deficient pachytene showed alterations in the number of MLH1 foci and the condensation of the sexual body. In addition, tamoxifen-inducible TCFL5 knockout mice showed, besides meiosis phenotype, alterations in the spermatids elongation process resulting in aberrant spermatids. Furthermore, TCFL5 deficiency increased spermatogonia maintenance genes (*Dalz, Sox2, and Dmrt1*) but also increased meiosis genes (*Syce1, Stag3, and Morc2a*) suggesting that the synaptonemal complex forms well, but cannot separate and meiosis does not proceed. TCFL5 is able to bind to the promoter of *Syce1, Stag3, Dmrt1*, and *Syce1* suggesting a direct control of their expression. In conclusion, TCFL5 plays an essential role in spermatogenesis progression being indispensable for meiosis resolution and spermatids maturation.

## Introduction

Spermatogenesis is a complex process that implies different cellular mechanisms to generate spermatozoa containing half of the genetic material of the precursor spermatogonia. It can be divided into three different stages: mitosis, meiosis, and spermiogenesis^1^. In the first step, spermatogonia, which is maintained by a series of mitotic divisions in a proliferative phase, differentiate to primary spermatocytes^2^. In this process, several extrinsic and intrinsic factors act together to maintain the correct self-renewal or promote differentiation to spermatocytes. Alterations in these pathways lead to early fertility issues^3,4^. The next phase comprises two consecutive meiotic divisions where primary spermatocytes undergo secondary spermatocytes during meiosis-I and, then generate haploid spermatids as a result of the meiosis-II^5^. The deregulation of meiosis leads to a meiotic arrest and aberrant spermatogenesis that in most cases produces infertility^6,7^. Finally, during spermiogenesis, haploid round spermatids suffer a complex structural transformation to become spermatozoa, comprised of the following steps: (1) formation of the acrosome, (2) condensation of the nucleus, (3) development of the sperm flagellum, (4) reorganization of cellular organelles, and (5) reduction of the cytoplasm^8^.

The meiotic process is the most important phase of spermatogenesis. Here, diploid cells give rise to haploid cells suffering two consecutive divisions following one round of replication and recombining their DNA. The main step involves the prophase-I of meiosis-I. In prophase-I homologous chromosomes recombine and generate genetic diversity. This phase is well known and is divided into 5 stages: (1) leptotene, where chromosome condensation is produced; (2) zygotene, where the pairing of homologous chromosomes starts and synaptonemal complexes, a multi-protein structure that held homologous chromosomes together^9^, are formed; (3) pachytene, where genetic material derived from paternal and maternal homologous chromosomes is exchanged by recombination of sister chromatids, (4) diplotene, where synaptonemal complexes disassemble, chromosomes separate except at the chiasmata sites, and (5) diakinesis, where chromosomes condense and the nuclear envelope disintegrates^10^. Formation of DNA double-strand breaks (DSBs), homologous recognition, synapsis, and meiotic recombination are necessary for the correct progression of prophase-I. At the end of the meiotic prophase-I, the four sister chromatids are visible, the nuclear envelope disappears, and chromosomes arrange on the metaphase plate. The resulting secondary spermatocytes proceed to the second meiotic division, this time without DNA synthesis, resulting in haploid round spermatids.

Transcription factors (TFs) are key players in the control of spermatogenesis. Specifically, some members of the basic Helix Loop Helix (bHLH) family of TFs are well known as a fundamental proteins to the correct development of gametes^11,12^. *Transcription Factor Like 5* (TCFL5) is a bHLH scarcely studied, first described in 1998 in human testis, specifically at the diplotene stage of primary spermatocytes^13^. Since then, few studies have reported its role during the spermatogenesis process. In mice, TCFL5 binds to the non-canonical E-box region CACGCG of Calmegin (*Clgn*) promoter^14^, a chaperon required for sperm mobility and adhesion to zona pellucida^15,16^. In addition, TCFL5 was found expressed in early and late elongating spermatids co-localizing with α-tubulin and centrioles, respectively. It is not expressed in mature spermatozoa^17^. TCFL5 is also described as a partner of MEIG1, a protein involved in spermiogenesis^18^. Recently, it has been related to the lack of TCFL5 expression in human testicular tumors with a pre-meiotic origin^19^.

To investigate the role of TCFL5 in spermatogenesis, we generated two TCFL5 knockout mouse models. The complete TCFL5 null mice model, using *Sox2* promoter-controlled CRE recombinase, led to infertility in male mice. The phenotypic analysis of this model indicates that TCFL5 is necessary for meiosis progression. Besides, the tamoxifen-conditional knockout model shows that TCFL5, in addition to the above, also has a role during the spermiogenesis process. Gene expression analysis suggests that transcription factor TCFL5 could control important genes in spermatogenesis affecting mitosis, meiosis and spermiogenesis. Our data demonstrate the essential role of TCFL5 in spermatogenesis progression being indispensable for meiosis resolution and spermatids maturation.

## Material and methods

### Tcfl5 knockout mice

Conditional TCFL5 LoxP system (TCFL5^fl/fl^) mice were created by Cyagen Bioscience using standard procedures. Briefly, a recombinant vector for TCFL5 flanking exon 4 with a LoxP system was transfected in embryonic stem cells (ESC). Selected ESCs were microinjected in blastocysts, followed by chimera production. Germline transmission was confirmed after breeding with wild-type mice. To remove exon 4, two strategies were carried out: (1) Ubiquitous TCFL5 knockout mice were generated crossing with SOX2^cre/cre^ mice (B6.Cg-Edil3^Tg(Sox2-cre)1Amc^/J, Jackson Laboratory #008454). After Cre deletion, a constitutive TCFL5 knockout mice line was established; (2) Conditional TCFL5 knockout mice dependent on tamoxifen administration were generated crossing with CAGGCRE-ER™ mice (B6.Cg-Tg(CAG-cre/Esr1*)5Amc/J, Jackson Laboratory #004682). Tamoxifen-inducible cre-mediated recombination system is driven by the chicken beta actin promoter/enhancer coupled with the cytomegalovirus (CMV) immediate-early enhancer. Cre recombinase is fused to a G525R mutant form of the mouse estrogen receptor being restricted to cytoplasm and, only after tamoxifen treatment, it access to the nucleus.

All animals were allowed free access to water and food. Animals were euthanized by CO_2_ inhalation at the end of every experiment. All animal care procedures used in this study were carried out in accordance with ARRIVE guidelines. All animal procedures were performed in strict accordance with the European Commission legislation for the protection of animal used purposes (2010/63/EU). The protocol for the treatment of the animals was approved by the Comité de Ética de la Dirección General del Medio Ambiente de la Comunidad de Madrid, Spain and was supervised by the Ethics Committee of CBMSO (Madrid, Spain).

### Histological analysis

Testes and epididymis from 8-week-mice were fixed in 4% phosphate-buffered formalin (pH 7.4) and embedded in paraffin. 3 μm paraffin-embedded sections were stained with Hematoxylin–Eosin (HE) or Periodic Acid-Schiff (PAS) as described previously. Images were taken with an Axioskop2 Plus (Zeiss) coupled to DMC6200 camera (Leica).

### Immunofluorescence

Testes from 8 week-mice were decapsulated and seminiferous tubules were fixed and processed for spermatocyte spreading by the drying-down technique previously described^20^, or processed for squashing as previously reported^21^. Spread spermatocytes and squashed seminiferous tubules slides were rinsed three times in PBS and blocked with 2% Bovine Serum Albumin (BSA) in PBS for 20 min at room temperature (RT). After blocking, slides were incubated overnight at 4 °C with anti-TCFL5 (Sigma), anti-SYCP3 (Cell Signalling), anti-SYCP1 (Abcam), anti-H1t (Cell Signalling), anti-γH2AX (Cell Signalling), anti-MLH1 (Pharmingen), anti-Pan-cadherin (Cell Signaling) and anti-Tubulin (Cell Signalling) primary antibodies. Then, slides were washed in PBS and incubated for 1 h at RT with their corresponding secondary antibodies against rabbit, mouse, and guinea pig IgGs conjugated with Alexa 555, Alexa 594, and Alexa 488 (Molecular Probes). For actin and flagellar mitochondria immunodetections Alexa 488 Phalloidin (Thermo Fisher) and Mitotracker Red CM-H 2 Xros (Thermo Fisher) were used, respectively. Finally, cells were stained with 1 μg/ml DAPI and mounted using ProLong Glass Antifade Mountant (Thermo Fisher Scientific). Images and stacks were taken in sCMOS camera on a Zeiss Axiovert microscope and analyzed using ImageJ (NIH) and Adobe Photoshop CS5.1 software.

### TUNEL assay

Apoptotic cells were determined using Click-iT™ Plus TUNEL assay kit (Thermo Fisher). 3 μm formalin fixed paraffin-embedded sections of testis were stained according to the manufacturer’s instructions. Images and stacks were taken in sCMOS camera on a Zeis Axiovert microscope and analyzed using ImageJ (NIH) and Adobe Photoshop CS5.1 software.

### Immunohistochemistry and immunohistofluorescence

Testes from 8 week-mice were fixed in 4% phosphate-buffered formalin (pH 7.4) and embedded in paraffin. 3 μm paraffin-embedded sections were incubated with antibodies for immunohistochemistry (IHC) or immunohistofluorescence (IFC): anti-TCFL5 (Sigma), anti-SYCP3 (Cell Signalling) and anti-panCadherin (Cell Signalling), and their corresponding secondary antibodies conjugated with HRP (Envision + Dual link System HRP, Dako) for IHC, or Alexas 594 and 488 (Molecular Probes), for IFC. Finally, IHC sections were developed using DAB solution (Liquid DAB + substrate chromogen system, DAKO K3468), counterstained with Hematoxylin and images were taken with a sCMOS camera on a Zeis Axiovert microscope. IFC sections were counterstained with 1 μg/ml DAPI and images were taken with a LEICA DMD108 Digital Microimaging Device (Leica Microsystems).

### Western blot analysis

Protein was obtained from 8-weeks-old mice testes using RIPA cell lysis buffer. Protein concentration was determined using Pierce BCA Protein Assay Kit (Pierce Biotechnology) according to the manufacturer’s instructions. Protein was resolved on 10–16% SDS-PAGE gel and transferred to nitrocellulose membrane (BioRad). Membranes were blocked in 5% BSA in Tris-Buffered-Saline with 0.1% Tween20 (TBS-T). For specific protein detection, membranes were incubated with anti-TCFL5-E1 (custom made antibody against CAGPDGAPEARAKPAVR peptide of exon 1, GenScript), anti-SYCP3 (Cell Signaling) and anti-HSP90 (Santa Cruz) in 5% BSA-TBS-T overnight at 4 °C. After washing the membrane, it was incubated with HRP-conjugated secondary antibody for 1 h at RT. Luminescence signal was detected in Amersham Imager 600 (GE Healthcare).

### RNA analysis

RNA was isolated from 8-weeks-old mice testes using TRIzol (Invitrogen) according to the manufacturer’s instruction. 1 μg of total RNA and sorted cells was reverse transcribed using High Capacity cDNA Reverse Transcription Kit (Applied Biosystems) following the manufacturer’s recommendation. The resulting cDNA was used both for PCR using GoTaq Flexi DNA Polymerase (Promega) or quantitative PCR (qPCR) using GoTaq 1PCR Master Mix (Promega) with specific primers (Supplementary Table 1), according to the manufacturer’s instructions. For qPCR, relative mRNA levels were calculated in accordance with the point where each curve crossed the threshold cycle (Ct) from ABI Prism 7900HT (Applied Biosystems), subtracting the Ct of the 18S housekeeping gene (∆Ct) according to the formula: 2^−(∆Ct problem−∆Ct control)^.

### Chromatin immunoprecipitation (CHIP) analysis

Seminiferous tubules were digested into a single-cell suspension as previously described^22^. Chromatin immunoprecipitation (ChIP) was performed as described^23^. Briefly, testis single-cell suspension was fixed, cross-linked, and lysed. The cross-linked cell chromatin was sheared by sonication using a Bioruptor Next Gen (Diagenode). Sheared chromatin was incubated with Dynabeads Protein G (Sigma), and anti-TCFL5 (Sigma). Protein–DNA complex was immunoprecipitated using QuadroMACS Separator (Miltenyl Biotec). DNA was isolated and analyzed by PCR and qPCR using specific oligonucleotides (Supplementary Table 1).

### Statistics

Statistical analysis was performed using GraphPad Prism v6.0 software (GraphPad Software, LLC). Results were expressed as means ± SEM (Standard Error of the Mean). Student’s t-test was used for differences significance, marked as *p > 0.05, **p > 0.01 and ***p > 0.001.

## Results

### TCFL5 expression and knockout generation

**Figure 1:**
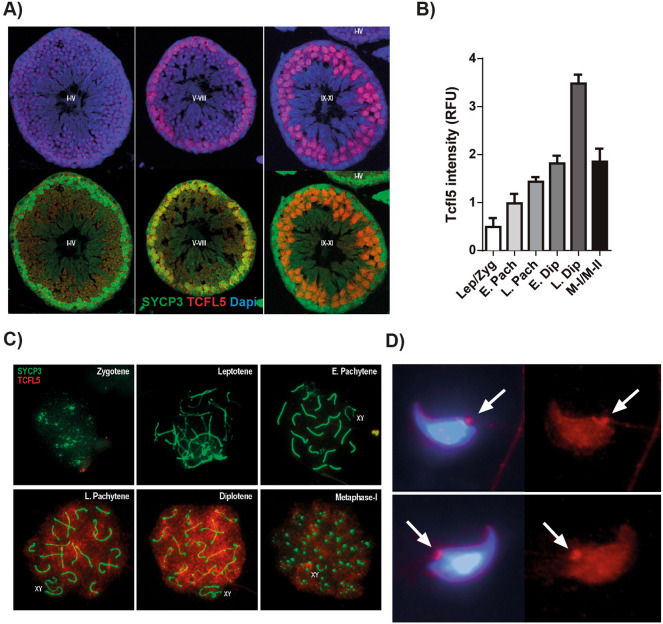
TCFL5 distribution during meiosis-I. (**A**) Immunohistofluorescence of seminiferous tubules stages I–IV, V–VIII and IX–X in TCFL5^+/+^ mice. TCFL5 (red), SYCP3 (green) and nucleus (Dapi, Blue) were detected. TCFL5 is expressed in spermatocytes but neither spermatogonia nor spermatids. (**B**) Quantification of TCFL5 expression in spread primary spermatocytes. TCFL5 is mostly expressed from late pachytene to diplotene. (**C**) Representative immunofluorescence of spread primary spermatocytes in TCFL5^+/+^ mice. SYCP3 (green), TCFL5 (red) and nucleus (Dapi, Blue) were detected. D) Representative immunofluorescence of spermatids. TCFL5 (red) and nucleus (Dapi, Blue) were detected.

TCFL5 expression has been described during the first meiotic prophase. In order to accurately study the effect of Tcfl5 deletion and infer its function, we first established the detailed distribution and expression levels of TCFL5 during male meiosis-I. For this purpose, we performed double immunostainings in both testis cross-sections and spermatocytes spread, of TCFL5 with SYCP3, a structural component of the axial/lateral elements of the synaptonemal complex that allows identifying the different meiotic-I stages. Immunofluorescence of TCFL5 in cross-sections of WT testis showed TCFL5 staining in stages V-VIII and IX-XI indicating it is expressed in spermatocytes but not in spermatogonia and spermatids (Fig. 1A). More in detail, the analysis of the expression level of TCFL5 in spread spermatocytes showed that TCFL5 expression begins during early pachytene and presents a significant increase at mid/late pachytene reaching its higher expression at diplotene distributed among the whole chromatin (Fig. 1B,C). Then, TCFL5 expression was gradually decreasing in the latter stages of the meiosis-I. In addition, no expression was detected in spermatids from immunofluorescence cross-section, however, testicular spread showed TCFL5 expression in spermatids concentrated at the base of the flagellum (Fig. 1D).

**Figure 2:**
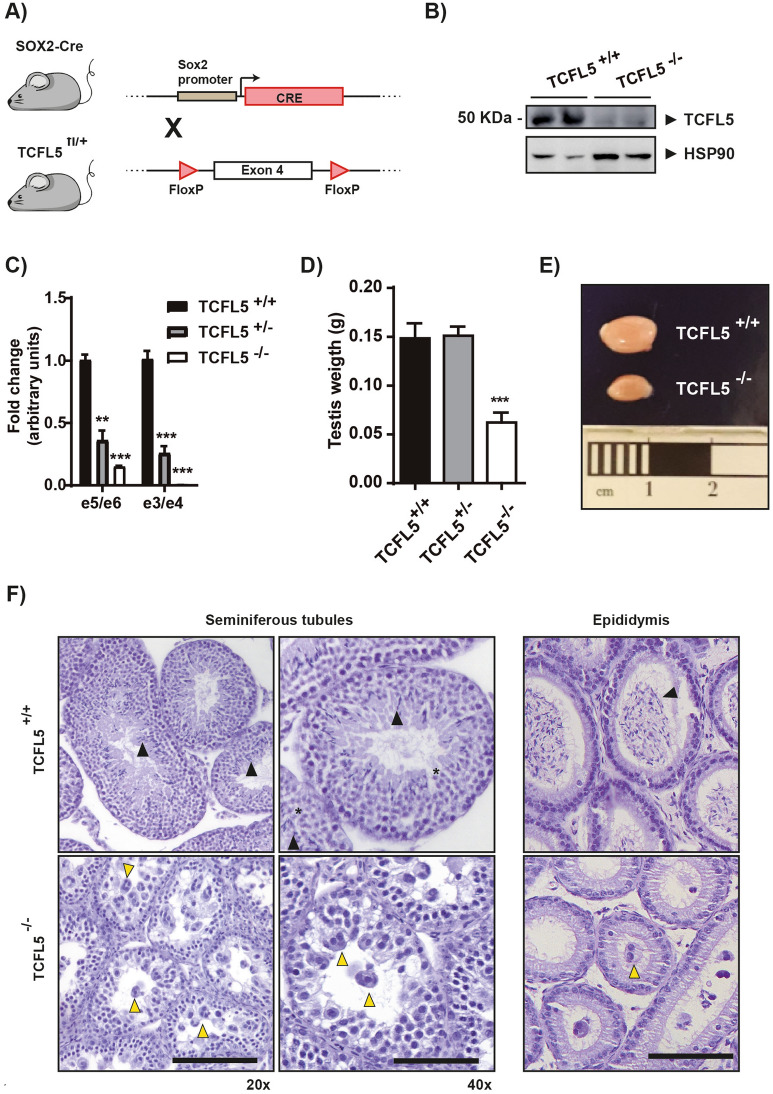
TCFL5^−/−^ mice alterations present in testis. (**A**) Scheme of TCFL5 knockout (TCFL5^−/−^) mice generation-strategy. TCFL5^fl/fl^ mice were crossed with SOX2-Cre mice to remove exon 4 and generate an aberrant transcript for TCFL5 from the first stage of development. (**B**) TCFL5 protein detection by WB. TCFL5^−/−^ mice reduce drastically TCFL5 expression. (**C**) *Tcfl5* mRNA expression determined by qPCR using two different primer pairs. TCFL5^+/−^ mice present half the expression of the TCFL5^+/+^ mice. TCFL5^−/−^ mice reduce drastically *Tcfl5* expression. (n = 8), t-test p < 0.01 (**), p < 0.001 (***). (**D**) Testis weights from TCFL5^+/+^, TCFL5^+/−^ and TCFL5^−/−^ mice. TCFL5^−/−^ present half the weight of the TCFL5^+/+^ while TCFL5^+/−^ do not show difference in testis weight. (n = 8), ***p < 0.001. (**E**) Testis sizes from TCFL5^+/+^ and TCFL5^−/−^. TCFL5^−/−^ present half the size of the TCFL5^+/+^. (**F**) Seminiferous tubules architecture and epididymis sections from TCFL5^+/+^ and TCFL5^−/−^ mice. TCFL5^+/+^ mice present no alteration and normal spermatids (black triangle). Abnormal morphology features with multinucleated rounded giant cells (yellow triangle) and no spermatozoa in the TCFL5^−/−^ mice. Scale bar 100 µm in a and b. Zoom 1.5X in a’ and b’.

To assess the function of *Tcfl5* during in vivo spermatogenesis, we generated a conditional TCFL5 knockout mouse using LoxP system, which was inserted flanking exon 4 (hereafter named TCFL5^fl/fl^). Exon 4 contains part of the bHLH domain so its deletion produces an aberrant and non-functional protein (Supplementary Fig. 1A). Then, two strategies were carried out to remove exon 4. First, we generated a constitutive Tcfl5 knockout crossing TCFL5^fl/fl^ mice to SOX2-Cre mice^24^ (Fig. 2A). Sox2 is expressed during the first stages of embryo development. After Cre deletion, pups were crossed to establish a Tcfl5 knockout mice line (hereafter named TCFL5^−/−^). TCFL5 protein detection confirmed the lack of detectable TCFL5 expression in null mice (Fig. 2B). Moreover, quantitative mRNA expression analysis of the testis demonstrates a complete reduction of e3/e4 expression and a strong reduction of e5/e6, although this was not complete in null mice (Fig. 2C). Heterozygous mice also showed a *Tcfl5* expression reduction indicating a gene dosage effect. The second strategy consisted of the generation of conditional Tcfl5 knockout mice dependent on tamoxifen administration in adult mice. To do this, TCFL5^fl/fl^ mice were crossed to Tx-Cre mice^25^ (hereafter named TCFL5-Tx) (Supplementary Fig. 2A). TCFL5-Tx mice were treated intraperitoneally with tamoxifen for 5 days. Exon 4 recombination was observed specifically in those Tcfl5-Tx mice treated with tamoxifen (Supplementary Fig. 2B). Analysis of the mRNA expression of testis showed that *Tcfl5* expression was, as expected, reduced in TCFL5-Tx mice after treatment (Supplementary Fig. 2C).

Recent database entries have shown several sequences for *Tcfl5* (Supplementary Table 2). According to this information, the mouse *Tcfl5* locus is composed of 7 exons: e1, e2, e3, e4, e4b, e5 and e6. There are at least 4 putative alternative mRNA transcripts: (1) *Tcfl5*_e6a (the one mostly described in the literature) composed by exons e1, e2, e3, e4, e5, and e6; (2) *Tcfl5*_*e6*b composed by exons e1, e2, e3, e4 and e6; (3) *Tcfl5*_*e4b* composed by exons e1, e2, e3 and e4b; (4) *Tcfl5*_*e5* composed by the exons e1, e3, e4 and e5 (Supplementary Fig. 1B). We addressed the expression of those Tcfl5 isoforms in testis. Transcript amplification using PCR primers annealing on exons 1 and 6 (e1/e6), and e1/e5 showed that the canonical isoform *Tcfl5*_*e6* was expressed in TCFL5^+/+^ mouse testis discarding *Tcfl5*_*e6*∆ and *Tcfl5*_*e5*. Moreover, TCFL5^−/−^ testes lack the full-length transcript, although they show an aberrant mRNA transcript related to exon 4 removal (Supplementary Fig. 1C). Further, amplification of adjacent exons confirmed that the only isoform expressed in testis is the canonical *Tcfl5*_*e6*. TCFL5^−/−^ testes exon pattern expression confirmed the presence of aberrant transcripts (Supplementary Fig. 1D).

### TCFL5 deficiency produces severe alterations in spermatogenesis

TCFL5^−/−^ mice were viable and did not show visible developmental abnormalities. However, we noticed reproduction problems in both TCFL5^+/−^ and TCFL5^−/−^ mice. After recording mice crosses, we observed that TCFL5^+/−^ mice were fertile, but the number of litters obtained was lower compared to TCFL5^fl/fl^ control mice crosses (Supplementary Fig. 3A). More importantly, TCFL5^−/−^ mice crosses did not produce any litter, suggesting a severe fertility alteration. In addition, the percentage of TCFL5^+/−^ crosses with pups were less (32%) than TCFL5^fl/fl^ crosses (93%) (Supplementary Fig. 3B). Examination of testes of 8-week-old mice revealed that TCFL5^−/−^ testis size was remarkably reduced compared to TCFL5^+/−^ and WT (Fig. 2D,E). Histological analysis of the testes revealed that TCFL5^−/−^ mice presented severe defects in the seminiferous tubules architecture. Cross-sections of TCFL5 null testes showed abnormal morphology features, including multinucleated round giant cells containing primary spermatocytes, and no spermatozoa in the lumen of the seminiferous tubules, which denotes compromised spermatogenesis during meiosis. TCFL5^−/−^ mice lacked spermatozoa in the epididymis and presented some abnormal multinucleated round giant cells (Fig. 2F). Furthermore, epididymis smear showed the complete depletion of sperm in TCFL5^−/−^ mice, indicating the reason for their sterility (Data not shown).

To confirm these results and to have a different insight of the role of TCFL5 in the spermatogenesis process, we delete TCFL5 in adult mice using TCFL5-Tx mice. 8-weeks-old TCFL5-Tx mice were treated intraperitoneally with tamoxifen for 5 days. Mice were sacrificed at 45 days and testes were analyzed. Interestingly, testis size and weight were also lower in treated mice with tamoxifen than in non-treated mice (Supplementary Fig. 2D,E). Moreover, histological analysis of testes showed that tamoxifen treatment of TCFL5-Tx mice induced alterations of the seminiferous tubules structure, similar to those observed in TCFL5^−/−^ testis. Most of the seminiferous tubules in these testes presented multinucleated round giant cells containing primary spermatocytes and a reduced number of elongated spermatids in the lumen (Supplementary Fig. 2F). Altogether, these data corroborate the role of TCFL5 in spermatogenesis progression.

### TCFL5 deficiency arrests spermatocytes in the late pachytene stage

**Figure 3:**
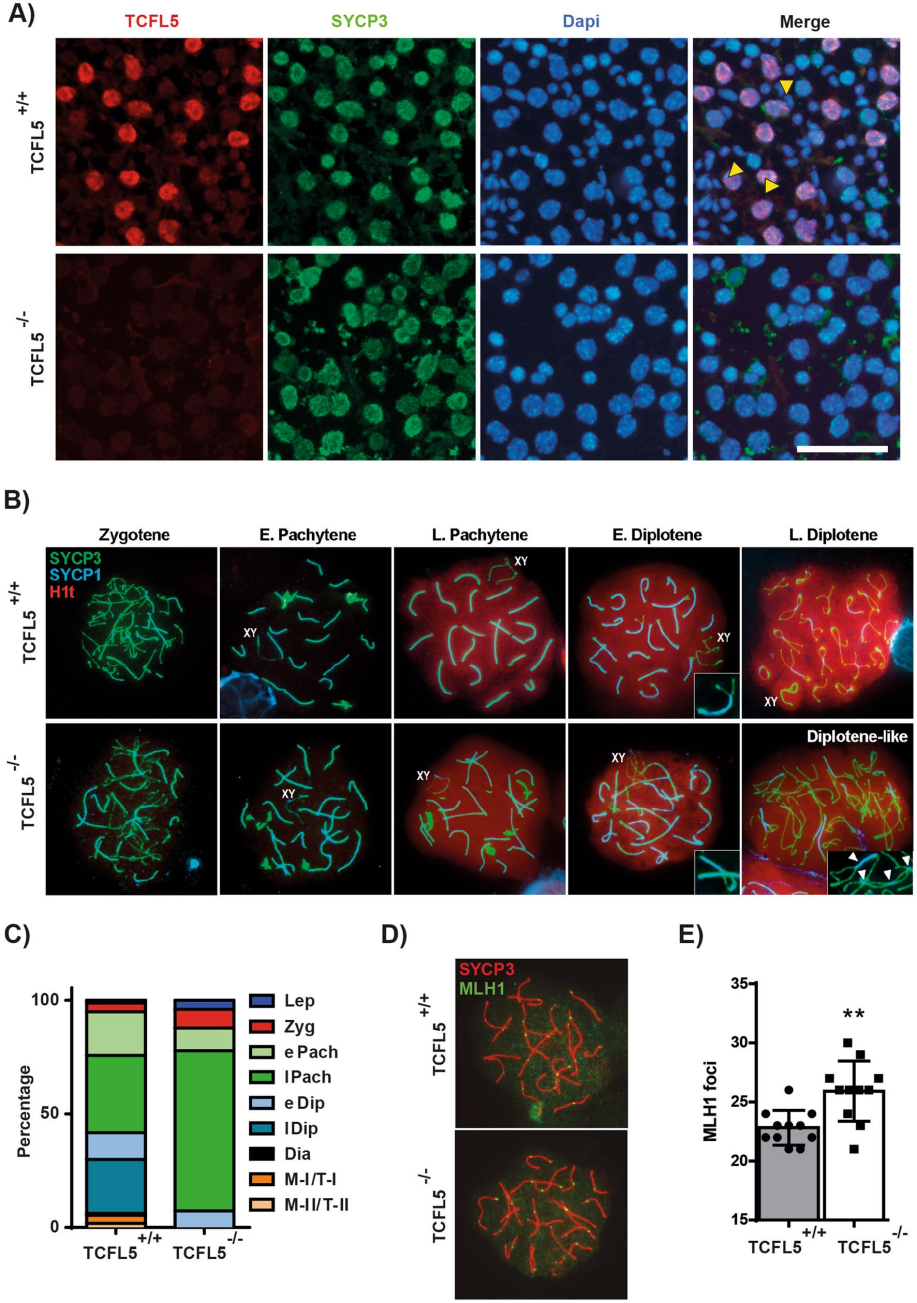
TCFL5 deficiency arrest cells in pachytene/diplotene transition. (**A**) Immunofluorescence of squashed seminiferous tubules in TCFL5^+/+^ and TCFL5^−/−^ mice. TCFL5 (red), SYCP3 (green) and nucleus (Dapi, Blue) were detected. TCFL5 was only expressed in TCFL5^+/+^ mice in cells that express SYCP3. Yellow triangles mark secondary spermatocytes and spermatids absence in TCFL5^−/−^ mice. Scale bar 40 µm. (**B**) Immunofluorescence of spread spermatocytes in TCFL5^+/+^ and TCFL5^−/−^ mice. SYCP3 (green), SYCP1 (blue) and H1t (red) were detected. SYCP3 staining appears disrupted in TCFL5^−/−^ mice. Scale bar 20 µm. (**C**) Quantification of meiotic stages in TCFL5^+/+^ (n = 3) and TCFL5^−/−^ mice (n = 3). (**D**) Immunofluorescence of spread spermatocytes in TCFL5^+/+^ and TCFL5^−/−^ mice. SYCP3 (green) and MLH1 (red) were detected. TCFL5^−/−^ mice presented more MLH1 foci. Scale bar 20 µm. (**E**) MLH1 quantification in TCFL5^+/+^ and TCFL5^−/−^ mice. (n = 8), **p < 0.01.

To study the effects of Tcfl5 null mice we first confirmed by immunofluorescence the absence of TCFL5 protein in TCFL5^−/−^ spermatocytes. As expected TCFL5 was only detected in the WT mice confirming the lack of TCFL5 expression in TCFL5^−/−^ testis. SYCP3 was detected in both WT and TCFL5^−/−^ primary spermatocytes suggesting that cells enter meiosis (Fig. 3A). In addition, SYCP3 protein expression by western blotting in testis extract from TCFL5^−/−^ did not show changes compared to WT (Supplementary Fig. 4). However, neither round nor elongated nucleus was detected in TCFL5^−/−^ mice suggesting the absence of secondary spermatocytes and spermatids (Fig. 3A), and in consequence, a meiotic arrest. These data demonstrate that TCFL5 deficiency impairs meiosis progression and suggests a possible arrest during meiosis-I.

To further investigate the meiosis-I arrest in TCFL5 null mice, we analyzed spermatocytes spreads the distribution pattern of two synaptic proteins: SYCP3 and SYCP1, a transversal filament protein of the central element of the synaptonemal complex (SC)^9^, and a specific testis marker of late prophase-I, the histone variant H1t. TCFL5^−/−^ chromosomes correctly synapse since zygotene and reach to pachytene stage. SYCP3 appears as thin lines (axial/lateral elements) at zygotene, while thicker lines are observed in pachytene corresponding to the SCs. SYCP1 is accumulated in the synapsed regions since zygotene, as occurs in WT. At pachytene, both markers colocalize at SCs and some SYCP3 extrachromosomal aggregations are observed during early-pachytenes in both conditions. However, at late prophase-I, when H1t is broadly detected throughout the chromatin, the abnormal SYCP3 aggregations remained in TCFL5^−/−^ spermatocytes. Besides, late-pachytene SYCP3 accumulates at the telomeres of each SC in WT spermatocytes but not in TCFL5^−/−^. Homologous unpairing marks diplotene onset and SYCP1 is lost from desynapsed regions. In WT diplotenes SCs became shorter due to chromosome condensation and SYCP3 accumulates at telomeres. However, TCFL5^−/−^ spermatocytes are not able to completely desynapse, chromosomes remain enlarged and there is no SYCP3 accumulation at telomeres. TCFL5^−/−^ spermatocytes remained in a *diplotene-like* stage (Fig. 3B).

Quantification analysis of the different meiosis stages in spermatocyte spreads confirmed that TCFL5^−/−^ spermatocytes arrest at early diplotene stage. Interestingly, TCFL5^−/−^ spermatocytes showed a significant accumulation at mid/late pachytene in comparison with WT (Fig. 3C). Altogether, these data confirm that meiosis progression is arrested at pachytene/diplotene transition in TCFL5^−/−^ male mice.

**Figure 4:**
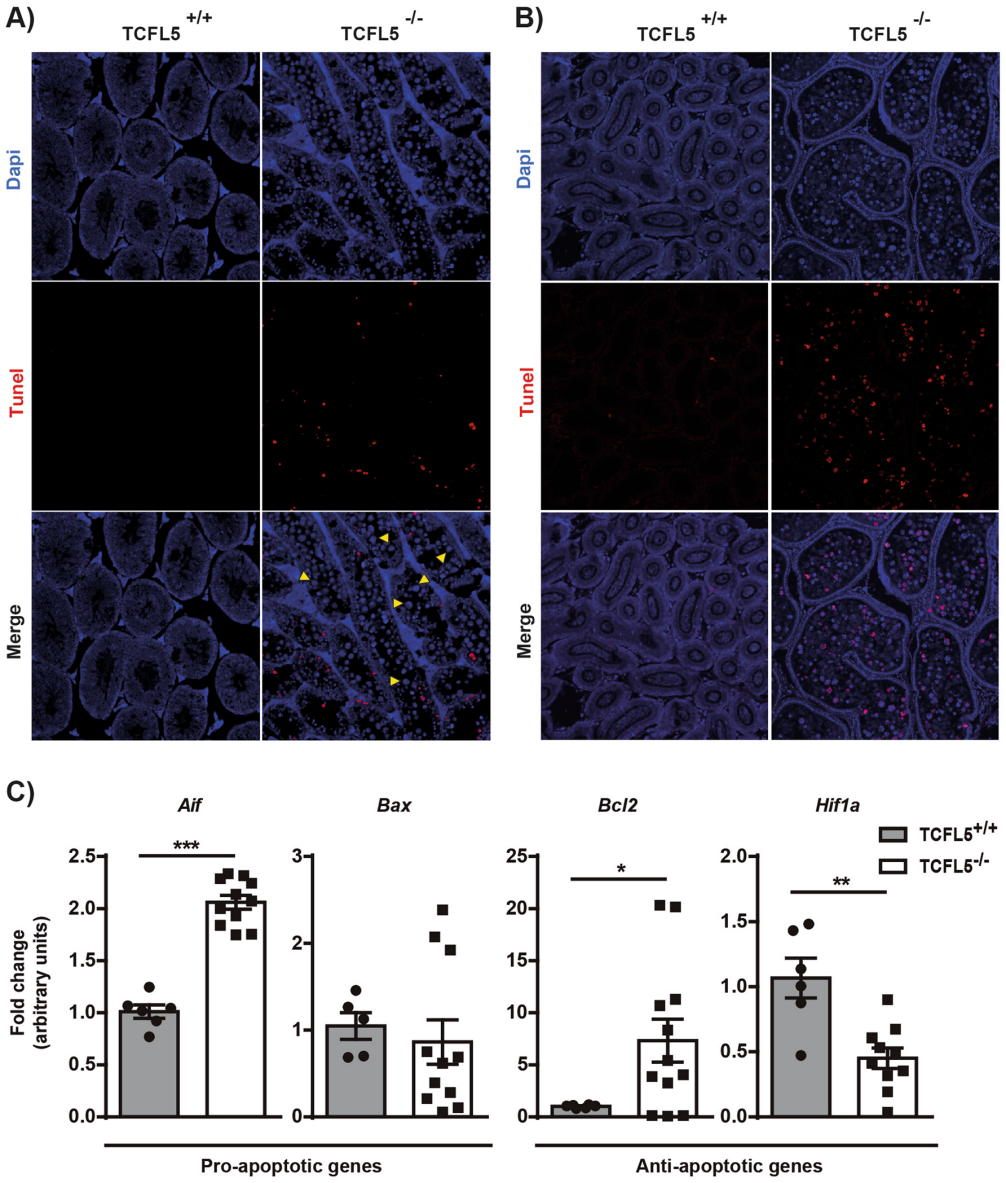
TCFL5 spermatocytes die in the epididymis. (**A**) TUNEL assay in seminiferous tubules cross-section of TCFL5^+/+^ and TCFL5^−/−^ mice. Apoptotic cells (red) and nucleus (Dapi, Blue) were detected. TCFL5^−/−^ mice present apoptotic cells in the seminiferous tubules. Yellow arrows show the live multinucleated round giant cells. (**B**) TUNEL assay in epididymis cross-section of TCFL5^+/+^ and TCFL5^−/−^ mice. Apoptotic cells (red) and nucleus (Dapi, Blue) were detected. TCFL5^−/−^ mice present apoptotic cells in the epididymis. (**C**) mRNA expression by qPCR of pro-apoptotic genes *Aif* and *Bax*, and anti-apoptotic genes *Bcl2*, and *Hif1a* in TCFL5^+/+^ and TCFL5^−/−^ mice. TCFL5 null mice present alterations in the expression of these genes. (n = 8), *p < 0.05, **p < 0.01, ***p < 0.001.

Multinucleated round giant cells were studied more in detail. SYCP3 immunohistochemistry staining revealed that the cells present in the cysts are SYCP3 positive indicating that these cells are in the prophase-I meiotic stage (Supplementary Fig. 5A). Specifically, the analysis of SYCP3 and H1t showed that cells contained in cyst are arrested in late-pachytene and diplotene-like stages (Supplementary Fig. 5B). In addition, pan-cadherin staining showed that these cells are included in the same cytoplasm revealing alterations in cyst conformation and/or cytokinesis process (Supplementary Fig. 5C). TUNEL assay showed that cells contained in cyst were mostly alive in seminiferous tubules (Fig. 4A). However, they enter into apoptosis when they reach the epididymis (Fig. 4B). In this sense, mRNA expression of both pro-apoptotic genes *Aif* and *Bax*, and anti-apoptotic genes *Bcl2* and *Hif1a* were altered in the seminiferous tubules extracts of null mice compared to WT mice (Fig. 4C). Altogether, these results suggest that cells contained in aberrant cysts were not able to finish the prophase-I stage and then activate the cell death pathway and spermatocytes are consequently eliminated by apoptosis in TCFL5^−/−^ mice.

### TCFL5 participate in the regulation of meiotic crossover formation

To further understand the possible causes of late-pachytene arrest, we performed immunolabelings on spread spermatocytes with γH2AX, a marker of unsynapsed chromosomes, recombination foci and sex chromosome inactivation, and MLH1, a marker of mature recombination nodules. As occurs in WT, TCFL5^−/−^ spermatocytes present a normal distribution of γH2AX marker. γH2AX is detectable at leptotene broadly localized throughout the nucleus and remained localized at unsynapsed chromosomes during early prophase-I. At pachytene onset, γH2AX marked the sex body chromatin and recombination foci along the SC marked with SYCP3. As pachytene progresses, γH2AX foci disappear being not detectable at diplotene. The number and pattern of these recombination foci seem not altered in TCFL5^−/−^ pachytenes. However, sexual body condensation of TCFL5^−/−^ late-pachytenes and diplotenes seem to be less condensed than in WT attending to the γH2AX area which is higher in TCFL5^−/−^ mice than WT (Supplementary Fig. 6A,B).

To analyse the final effect of the recombination process we performed double immunostaining of MLH1 and SYCP1. MLH1 is essential for crossover formation and in mice, MLH1 foci appear in mid-pachytene^26^. Surprisingly, the quantitative analysis of the MLH1 foci showed a significant increase in TCFL5^−/−^ pachytenes (27.62 ± 0.77) compare to WT (22.83 ± 0.44). This recombination foci accumulation could be due to a recombination misregulation in TCFL5^−/−^ spermatocytes (Fig. 3D,E).

### TCFL5 deficiency produces aberrant spermatids

**Figure 5:**
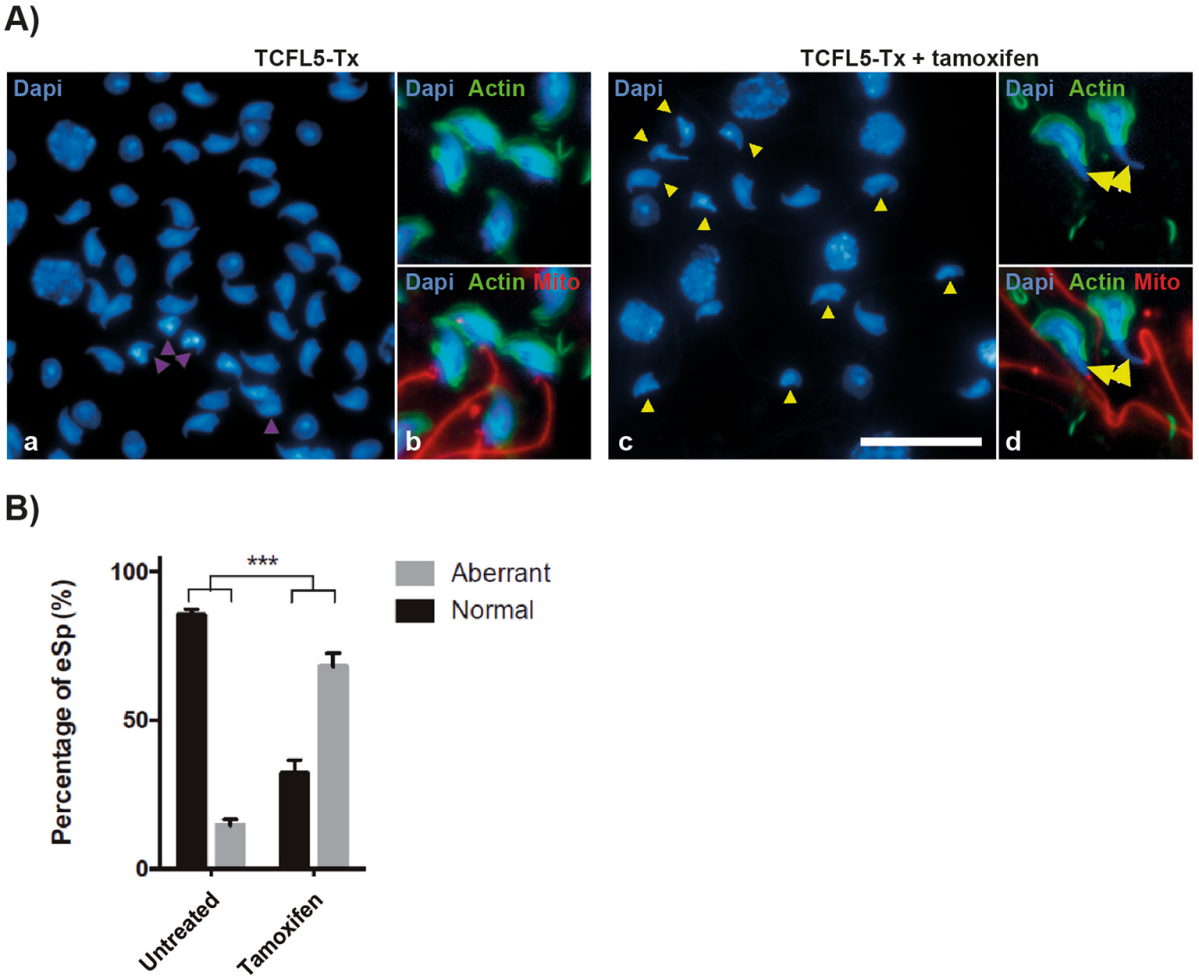
TCFL5-Tx treated with tamoxifen produced aberrant elongated spermatids. (**A**) Squashed eSp stained with DAPI in TCFL5-Tx non-treated and TCFL5-Tx treatesd with tamoxifen. TCFL5-Tx non-treated eSp present normal morphology (purple triangle), while most of TCFL5-Tx eSp present aberrant morphology (yellow triangle). Scale bar 20 µm. Actin (green), Mito (red) and nucleus (Dapi, Blue) were detected. (**B**) Percentage of elongated spermatids (eSp) in TCFL5-Tx non-treated and TCFL5-Tx treated with tamoxifen (TCFL5-Tx) mice. TCFL5-Tx non-treated mice produced an average of 14.6% aberrant eSp, while TCFL5-Tx treated an average of 67.9%, a 4.6-fold more aberrant eSp than TCFL5-Tx non-treated. (n = 4), t-test p < 0.001 (***).

Although TCFL5 null mice completely block meiosis and did not have secondary spermatocytes or spermatids, we observed another defect in TCFL5-Tx mice. TCFL5-Tx mice, contrary to TCFL5^−/−^, allow assessing if TCFL5 has a role in later stages of spermatogenesis. Interestingly, after tamoxifen treatment, we observed alterations in the morphology of elongated spermatids (Fig. 5A). In this sense, TCFL5-Tx mice produced an average of 4.5-fold more aberrant elongated spermatids than not treated mice (Fig. 5B). This significant increase suggests an important role of TCFL5 also in spermiogenesis.

### TCFL5 controls the expression of genes involved in different steps of spermatogenesis

To find potential candidates for TCFL5-related genes, we first obtained the most-*Tcfl5* correlated genes from the ARCHS4 web resource, a website that collects RNA-Seq data and analyzes them^27^ (Supplementary Table 3). Gene ontology and functional categories analysis^28,29^ showed that genes related to *Tcfl5* are mostly implicated in spermatogenesis, specifically in meiosis and spermiogenesis (Supplementary Fig. 7A,B). *Syce1* (synapsis), *Stag3* (cohesion), and *Morc2a* (chromatin-remodelling factor) were selected as representative genes of the list associated with meiosis. Interestingly, mRNA expression levels of all these 3 genes were increased in TCFL5^−/−^ mice, supporting the idea of a central role of TCFL5 in this process (Supplementary Fig. 7C). Moreover, the expression of genes implicated in the post-meiotic process, such as *Dcaf17*, *Spata16*, and *Tekt3* also correlated with that of *Tcfl5*. The mRNA analysis of correlated *Tcfl5* genes showed that TCFL5-Tx mice present lower expression levels of *Dcaf17* although this was not statistically significant (Supplementary Fig. 7D). In addition, we also wanted to study other important genes for spermatogenesis such as *Sox2*, *Dalz* and *Dmrt1.* All these genes were upregulated in TCFL5^−/−^ mice suggesting that TCFL5 could act as a negative regulator of these genes (Supplementary Fig. 7E).

To address if TCFL5 could regulate directly these genes more than only correlate, we search for the described TCFL5 binding motif (CACGCG) in the promoter of some of those genes^14^. We found that *Dazl*, *Dmrt1*, *Stag3*, *Syce1*, *Dcaf17*, and *Spata16* presented potential TCFL5 binding sites (Supplementary Fig. 7F), indicating a possible direct transcriptional control by TCFL5. Chromatin immunoprecipitation analysis confirmed that TCFL5 binds to the *Dazl*, *Dmrt1*, and *Syce1* promoters (Supplementary Fig. 7G,H) suggesting a direct control of these genes by TCFL5.

## Discussion

Although TCFL5 was initially identified in human testis^13^, its role during spermatogenesis has been scarcely studied. In humans, TCFL5 protein expression has been described in primary and secondary spermatocytes and round spermatids^19,30^. However, there is no consensus on its expression pattern in testis in mice. Siep M. et al. described TCFL5 expression in spermatocytes^14^, while Shi et al. found it in elongating spermatids^17^. They explain this difference by attending to the possible presence of several isoforms. Our analysis of the available genetics database indicates at least 4 potential different mRNA isoforms for *Tcfl5* in mice. However, our detailed analysis of the transcripts’ expression levels shows that only one mRNA isoform is present in the testicular extract. Furthermore, our immunofluorescence analyses confirm its presence in primary spermatocytes. Specifically, TCFL5 expression appears in the pachytene stage and presents the maximum expression in the diplotene stage. Reduced TCFL5 expression was detected in the following stages of spermatogenesis. This isoform and its expression agree with those described by Siep et al. Indeed, the main effect of TCFL5 knockout mice in spermatogenesis is found in pachytene/diplotene stages. Nonetheless, TCFL5 deficiency also affects spermatids. We described a lower and located expression of TCFL5 at the top of flagellum of spermatids concordant to Shi et al.^17^.

Regarding meiosis, TCFL5^−/−^ spermatocytes reach a diplotene-like stage, being unable to resolve it and producing cell arrest. SYCP3, SYCP1, H1t, and γH2AX expression revealed that TCFL5^−/−^ cells enter diplotene phase and MLH1 expression showed a higher number of the chiasma points. Even though chromosome desynapsis starts, cells are not able to complete the process. Furthermore, chromosomes at this stage do not correctly condense. These events lead cells to a diplotene-like stage, which cannot be resolved, and spermatocytes finally collapse and die. These results showed a complete block of meiosis impeding the spermatid generation. Supporting this, we found that *Tcfl5* is correlated to meiotic genes implicated in different aspects of the process, such as chromosome synapsis (*Syce1)*, sister chromatid cohesion (*Stag3)*, and chromatin remodelling (*Morc2a*). The expression of these genes in TCFL5 null mice was significantly increased. We found putative TCFL5 binding sites in *Stag3*, and *Syce1* promoters. However, we only confirmed by ChIP the direct control of *Syce1* by TCFL5. Altogether, these facts reveal that *Syce1* expression is specifically induced by TCFL5 whereas *Stag3* and *Morc2a* expression must be indirectly altered by TCFL5.

SYCE1 is a protein member of the synaptonemical complex^31,32^ and its deficiency produced meiosis arrest at the pachytene stages due to problems in chromosome synapsis^7^. In addition, STAG3 is a member of the meiotic cohesin complex allowing the correct chromosome segregation and is specifically expressed in spermatocytes associated to meiosis-specific synaptonemal complex^33^. In this case, STAG3 null mice produce a premature block in the zygotene stage of meiosis^34–36^. Our data indicate that these mice reach to pachytene stage with an apparently synaptonemal complex well formed. However, TCFL5 deficient mice present abnormal diplotene-like stages cells, which show the synaptonemal complex desynapsing in an anomalous way. All these data suggest that synaptonemal complex is correctly formed, but cells are not able to desynapse this complex and finally cells die. On the other hand, MORC2 is a member of the microrchidia (MORC) protein family, a chromatin-remodelling factor. *Morc2* present two paralogues: *Morc2a* and *Morc2b*. Their physiological functions are still unknown but it has been described that MORC2B is also essential for meiosis allowing chromosome recombination^37^. In this sense, we have observed more MLH1 foci in TCFL5 KO than in WT, indicating a higher number of recombination sites. These results show that TCFL5 acts as a transcription factor regulating genes implicated in meiosis. Although a comprehensive analysis of transcriptome data from TCFL5 knockout is necessary, we propose that TCFL5 could regulate the meiotic transcriptome network and act as an essential transcription factor in the meiosis process as other proteins have been defined such as STRA8 or MEIOSIN^38,39^.

In TCFL5 null mice, diplotene-like stages keep inside the cysts and reach the epididymis where cells definitively die. That could be related to an alteration in cysts formation. Germ cells form nests of associated cells before entering meiosis, in order to generate interconnected, synchronous cell groups, known as cysts^40^. Taking into account that alterations in cysts formation and maturation could lead to sterility^41,42^, TCFL5 could play an important role in the regulation of pre-meiotic cysts formation.

Previously, TCFL5 has been described to interact with MEIG1 and SPAG16 in spermatids^17,43^. Indeed, *Meig1* and *Tcfl5* appeared in our most correlated genes list. Both proteins, MEIG1 and SPAG16, have been described as important proteins for spermatids maturation and sperm motility^44–47^. The fact that TCFL5 interacts with proteins implicated in sperm flagellum formation suggests that TCFL5 could have another role besides as a transcription factor. Our studies have revealed that spermiogenesis is also affected. Due to the striking effect of TCFL5 in meiosis, it is impossible to observe spermatids defects in complete TCFL5 knockout mice. For this reason, conditional TCFL5-Tx mice are a better approach. The induction of TCFL5 deficiency by tamoxifen in mature mice affects all cells present in the testis. A great percentage of spermatids presented rare shapes in these mice. Our analysis of correlation showed that some proteins required for sperm maturation and motility such as *Dcaf17, Spata16*, and *Tekt3* are correlated to *Tcfl5*. In addition, *Dcaf17, and Spata16* present putative TCFL5 binding sites in their promoter. All these proteins produce spermatids arrest, rare sperm shape, and infertility syndrome in humans^48–52^ suggesting a possible explanation for the spermatid phenotype of TCFL5 null mice. Along these lines, TCFL5 has been described as a regulator of *Clgn* gene, a chaperone expressed in spermatogenesis^14^. However, null mice for this protein do not have visible defects in spermatogenesis but the resulting sperm is not able to adhere to zona pellucida. In contrast to our results, it has been recently described that TCFL5^+/−^ mice were infertile due to abnormal spermatids, and thus those authors were unable to generate, TCFL5^−/−^ knock out mice^53^. The discrepancies with our results may lie in the genetic mechanism used to delete the gene. Nonetheless, we observed a similar defect in spermatids in TCFL5-TX mice.

Interestingly, germ cell maintenance gen *Sox2*^54^, *Dmrt1* and *Dalz* are increased in TCFL5 knockout mice. In this regard, we observed that TCFL5 negatively regulates SOX2 in human tumor cells^55^. Thus, it is possible that the constant expression of Sox2 blocks meiosis activating germ cell maintenance signals. Indeed, *Dmrt1* is also increased in TCFL5^−/−^ mice and it has been described to be necessary for mitosis maintenance and its loss leads to meiosis entry^56,57^. Moreover, TCFL5 and DMRT1 have been reported to have an opposite expressions^19^ and we found that TCFL5 binds to *Dmrt1* promoter, suggesting that TCFL5 could be a transcriptional repressor of its expression. In agreement with this repressive role of TCFL5, a comprehensive functional enrichment analysis of male infertility, both in mouse models and in humans has found increased levels of *Tcfl5*^58^. Although this result may result contradictory, these models are related to *Etv5*, *Pou3f1*, and *Bcl6b* genes which are implicated in the spermatogonial stem cell renewal, being these mice sterile due to a loss of spermatogonia^59,60^. Thus, *Tcfl5* induction could be related to the lack of mitotic maintenance signals that would repress *Tcfl5* expression. *Dazl* is considered a master regulator of spermatogenesis and it is also induced by TCFL5 deficiency and TCFL5 also bound to its promoter. *Dazl* is required for correct progression through all processes, being one of its functions the spermatogonial stem cells maintenance^61^.

Here, we described the phenotype caused by TCFL5 deficiency, affecting mouse spermatogenesis. TCFL5 knockout results in azoospermia due to an arrest in the first meiotic prophase at the diplotene stage and conditional TCFL5-Tx results in the generation of abnormal spermatids. Altogether, our results demonstrate that TCFL5 is an essential transcription factor in those processes, involved in both meiosis resolution and spermatids maturation. Thus, TCFL5 binds to the promoter of some important genes in those processes suggesting a direct control of their expression, either inhibiting spermatogonia maintenance genes or activating meiosis genes and may play a master switch role. Overall, TCFL5 phenotype in spermatogenesis could be explained by attending to all these effects. Thus, TCFL5 may be a master regulator controlling several key steps in spermatogenesis.

## Supplementary Information

Supplementary Information 1.

Supplementary Information 2.

## Data Availability

The original contributions presented in the study are publicly available. Mice Tcfl5 sequences used in this study are deposited in NCBI (Accession number: 277353, NM_178254.3, BC108399.1, XM_006500653.1, AK132721.1, AY234363.1 and XM_006500652.1), Ensemble (Accession number: ENSMUST00000131881, ENSMUST0000037877, ENSMUST00000131881, ENSMUST00000161425), Vega (Accession number: OTTMUSG00000016345, OTTMUST00000039295, OTTMUST0000086954) and Uniprot (Accession number: Q32NY8, Q3V133, Q810E9, ESQ5K1) databases. Functional categories, gene ontology and correlation analysis in this database was performed 2020/04/15 and can be found here: ARCHS4 website, https://maayanlab.cloud/archs4/. Original gels/blots are available in Supplementary Fig. 8.
