# Supplementary material for: TCFL5 deficiency impairs the pachytene to diplotene transition during spermatogenesis in the mouse
Source: Sci Rep. 2022 Jun 29;12:10956. doi: 10.1038/s41598-022-15167-w (PMC9242989; doi:10.1038/s41598-022-15167-w)
Supplement: Supplementary file 1 — Supplementary Information 1. [file 41598_2022_15167_MOESM1_ESM.docx]

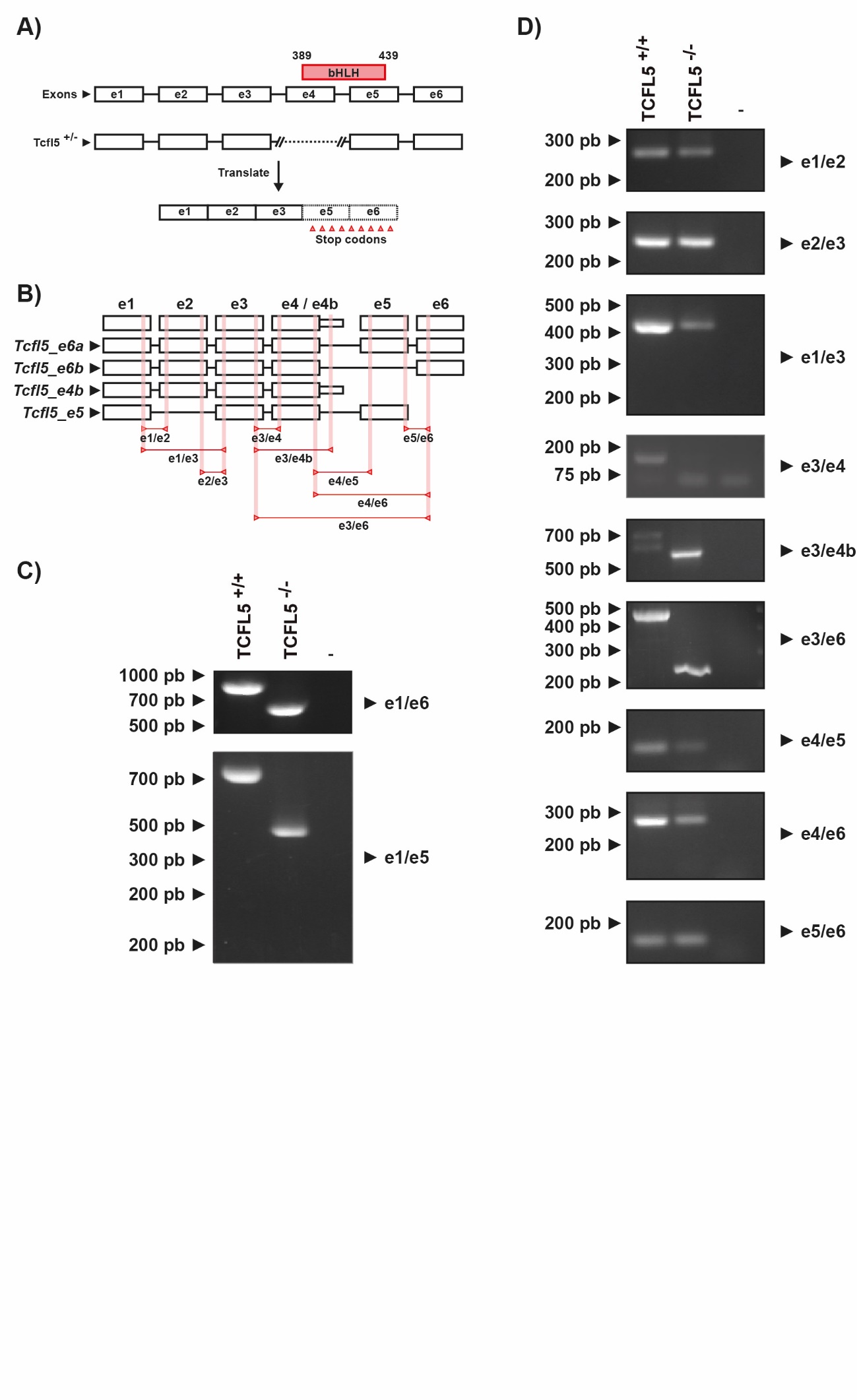


**Supplementary Figure 1.** ***Tcfl5* expression in TCFL5^-/-^ mice. A)** Scheme of TCFL5 knockout mice generation-strategy. Removal of exon 4 produces an aberrant transcript. **B)** Tcfl5 isoforms described in databases and primers used. **C)** Complete *Tcfl5* transcripts detected by PCR. TCFL5^-/-^ mice express an aberrant transcript resulting from exon 4 removal. **D)** Exon junctions for *Tcfl5* transcript in TCFL5^+/+^ and TCFL5^-/-^ mice. Exon junctions that compromise exon 4 are altered in TCFL5^-/-^ mice.

**Supplementary Figure 2. TCFL5-Tx treated with tamoxifen produced similar phenotype.** **A)** Scheme of TCFL5 tamoxifen conditional (TCFL5-Tx) mice generation-strategy. TCFL5^fl/fl^ mice were crossing with Cre-ER^TM^ mice to remove exon 4 and generate an aberrant transcript for TCFL5 after tamoxifen treatment. **B)** DNA recombination in TCFL5-TX mice. Exon 4 is partial or complete removed after tamoxifen treatment. **C)** *Tcfl5* mRNA expression determined by qPCR. TCFL5-Tx mice reduce *Tcfl5* expression after tamoxifen treatment. (n = 7), t-test p < 0.05 (*). **D)** Testis average weight from TCFL5-Tx mice with or without tamoxifen treatment. Testes weight was reduced in TCFL5-Tx mice treated with tamoxifen. **E)** Testis size in TCFL5-Tx mice with or without tamoxifen treatment. Testes size was reduced only in TCFL5-Tx mice treated with tamoxifen. (n = 7), t-test p<0.001 (***). **F)** Seminiferous tubules architecture from TCFL5-Tx mice with or without tamoxifen treatment. Tubules seminiferous architecture present abnormal morphology features with multinucleated rounded giant cells and no spermatozoa. Scale bar 100 µm in a and b. Zoom 1,5X in a’ and b’.


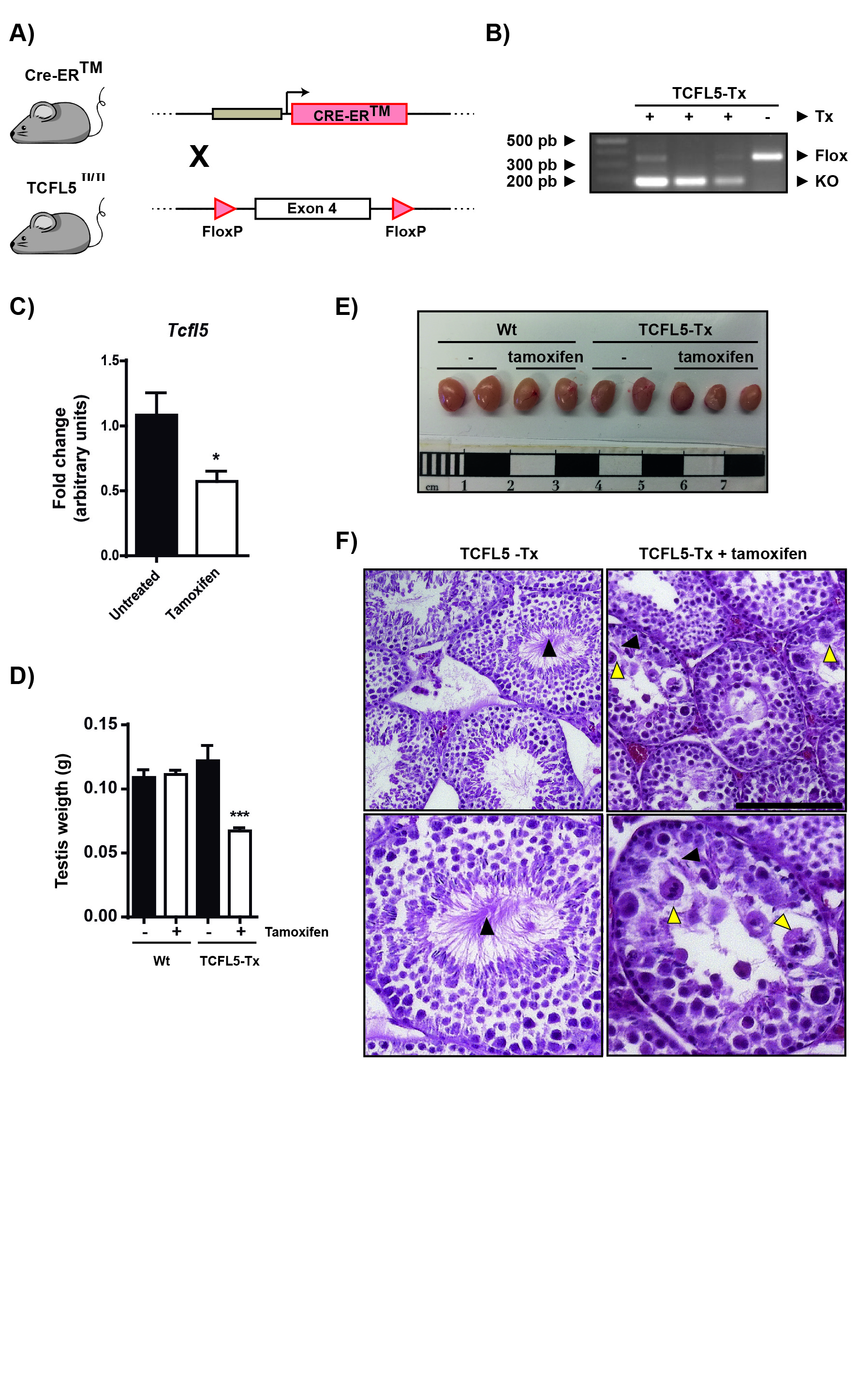

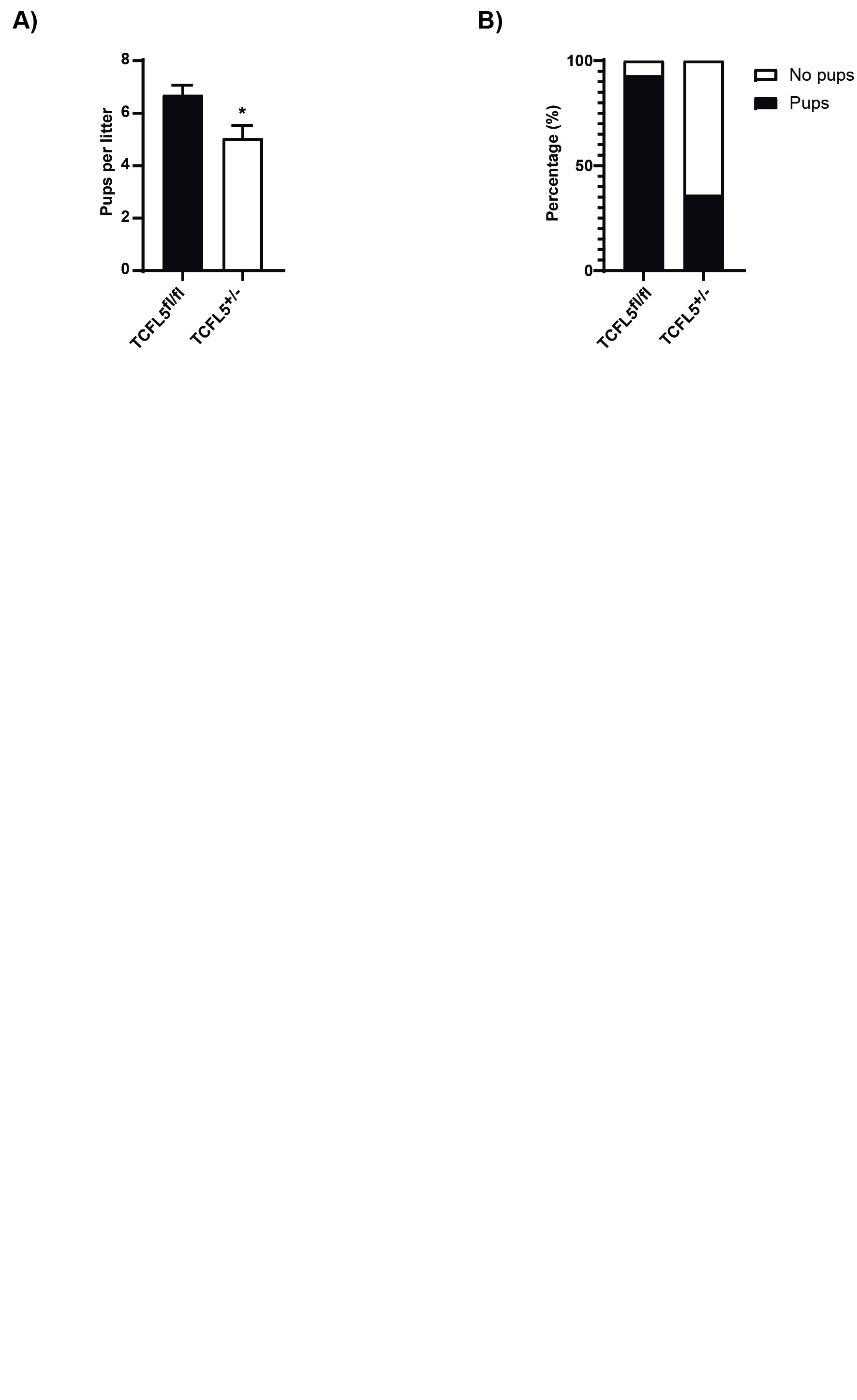


**Supplementary Figure 3. Heterozygous mice present fertility defects. A)** Pups per litter from TCFL5^fl/fl^ and TCFL5^+/-^ crosses. (n = 20), t-test p < 0.05 (*). **B)** Percentage of crosses with pups from TCFL5^fl/fl^ and TCFL5^+/-^ mice.

**Supplementary Figure 4. TCFL5 null mice does not present alteration in SYCP3 expression.** SYCP3 protein detection by WB. To the right, WB image. HSP90 was used as a control. To the left, WB quantification SYCP3 bans of 2 independent mice or experiments.


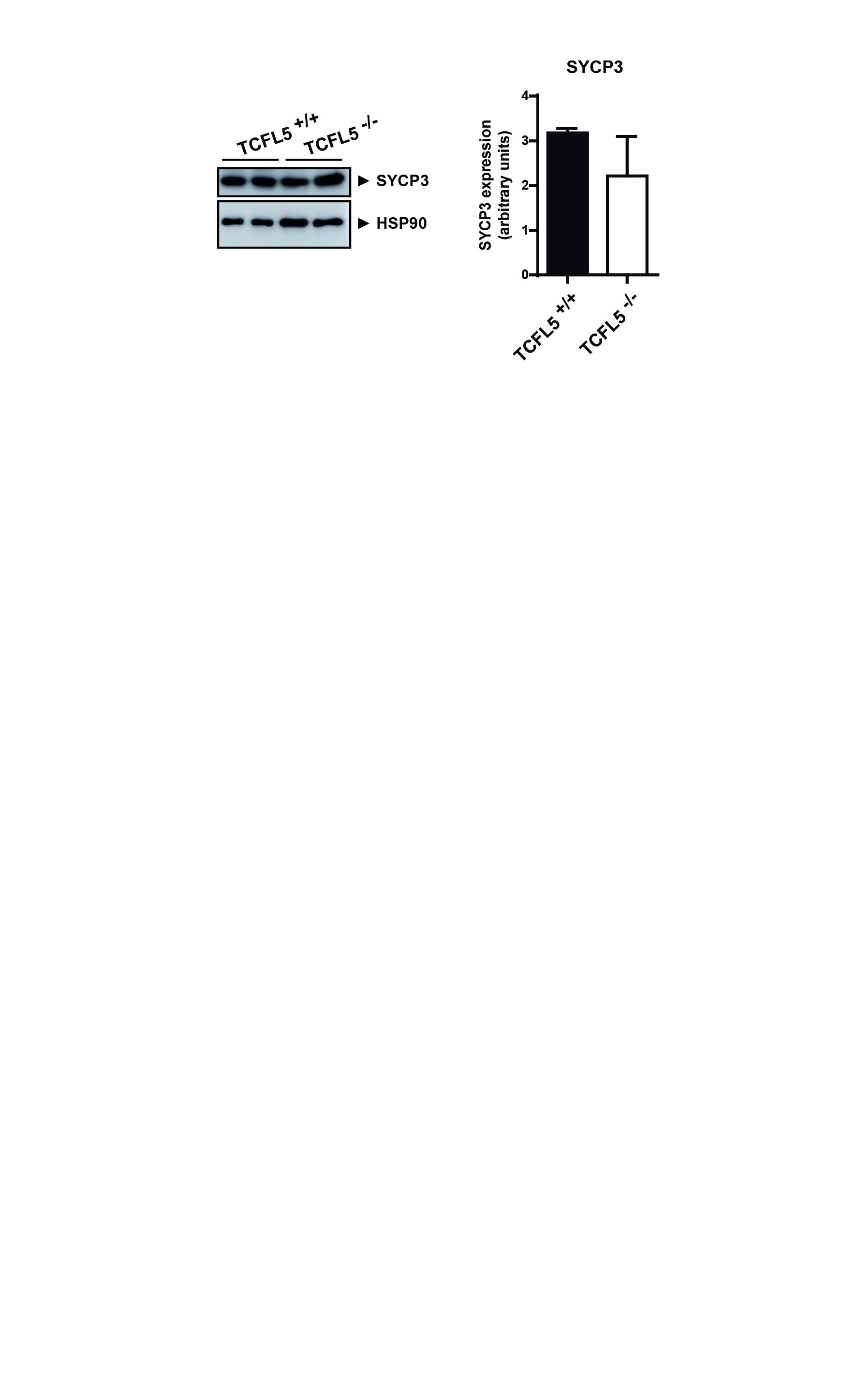


**Supplementary Figure 5. TCFL5^-/-^ cyst contain spermatocytes. A)** SYCP3 immunohistochemistry of seminiferous tubules in TCFL5^+/+^ and TCFL5^-/-^ mice. Multinucleated rounded giant cells of TCFL5^-/-^ express SYCP3. **B)** Multinucleated rounded giant cells immunofluorescence of spread seminiferous tubules in TCFL5^-/-^ mice. Sycp3 (green), γH2AX (red) and nucleus (Dapi, Blue) were detected. Scale bar 20 μm. **C)** Pan-Cadherin immunohistochemistry of seminiferous tubules in TCFL5^+/+^ and TCFL5^-/-^ mice. Multinucleated rounded giant cells of TCFL5^-/-^ are contained in the same membrane.


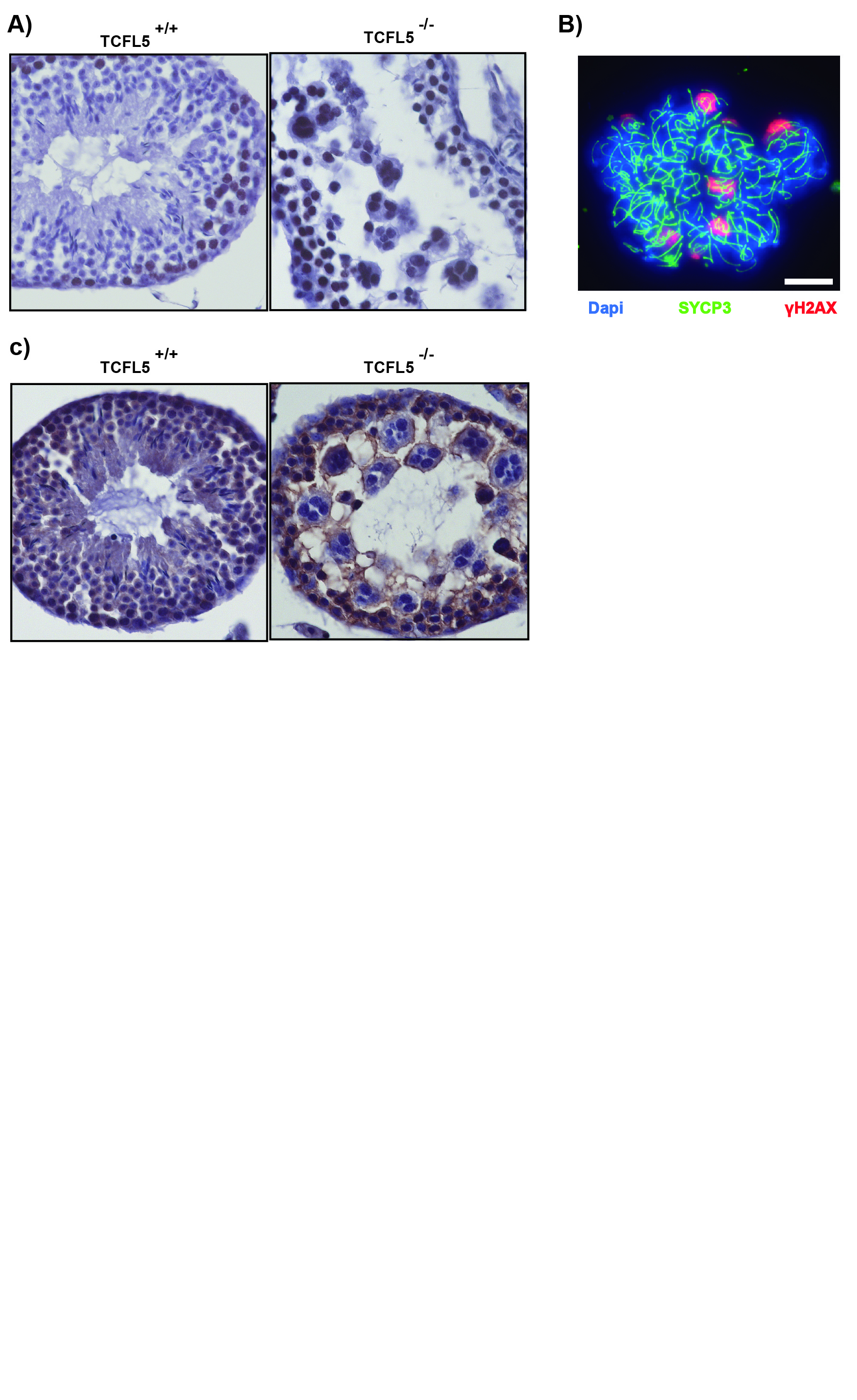

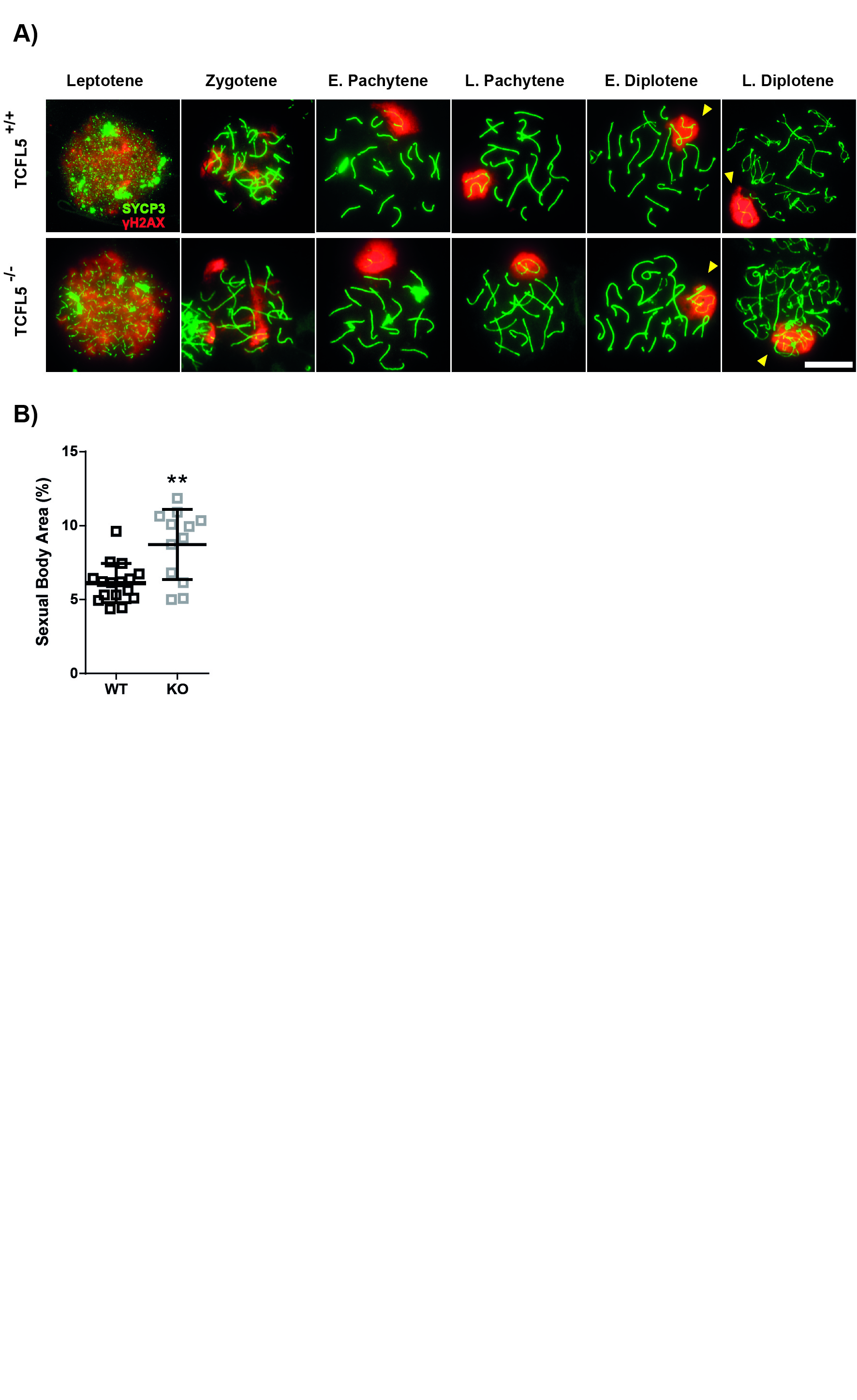


**Supplementary Figure 6.** **A)** Immunofluorescence of spread spermatocytes in TCFL5^+/+^ and TCFL5^-/-^ mice. SYCP3 (green) and γH2AX (red) were detected. Yellow arrow show the sex body. Scale bar 20µm. **B)** Quantification of γH2AX expression.


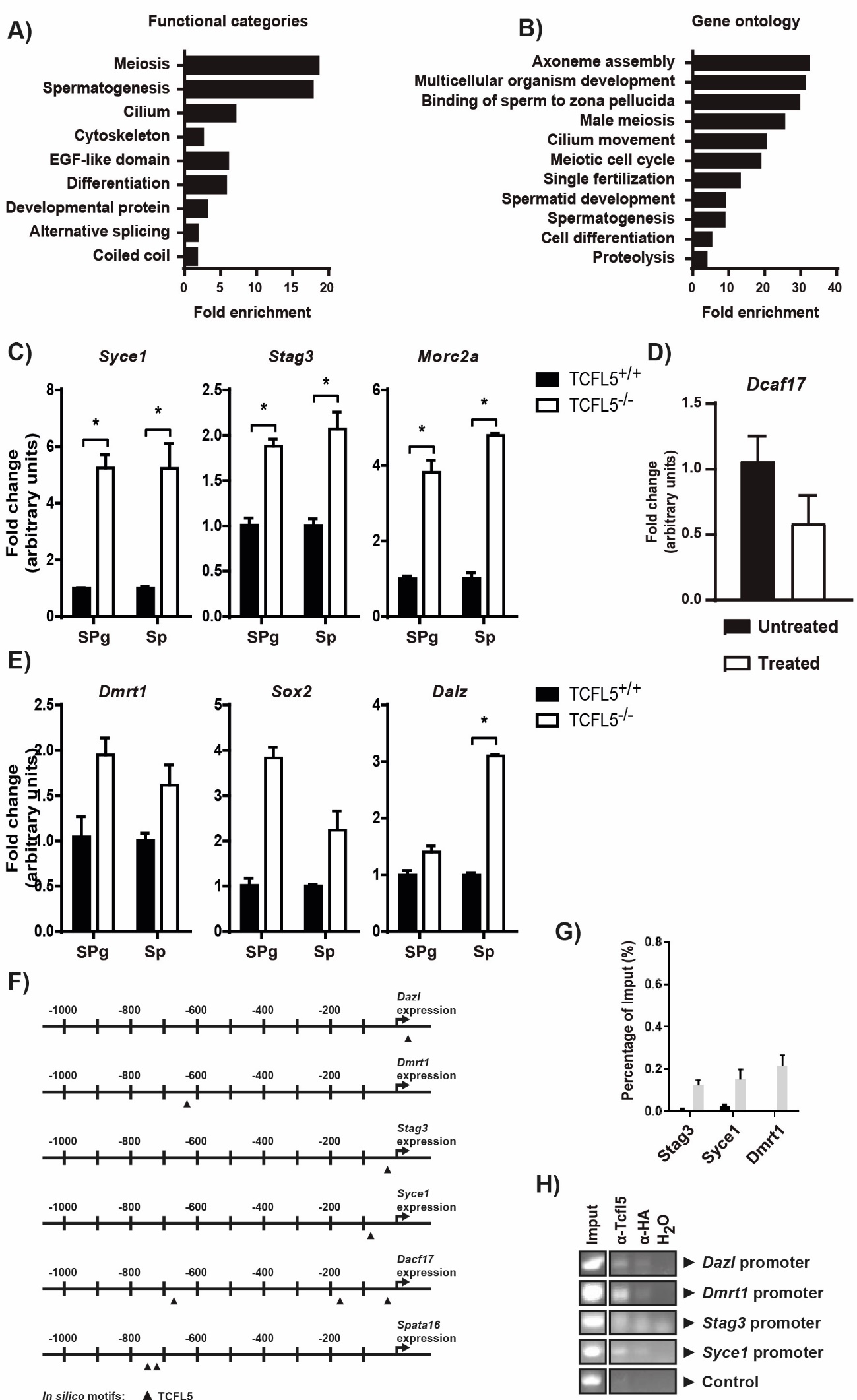


**Supplementary Figure 7.** ***Tcfl5* correlated with genes implicated in spermatogenesis.** **A-B)** Functional categories and Gene ontology analysis from the top 100 most *Tcfl5*-correlated genes. **C)** mRNA expression by qPCR of *Morc2a*, *Stag3* and *Syce1* in TCFL5^+/+^ and TCFL5^-/-^ mice. TCFL5 null mice present lower expression of *Morc2a*, *Stag3* and *Syce1* than TCFL5 wild type mice. (n = 8), t-test p<0.001 (***). **D)** mRNA expression by qPCR of *Dcaf17* in TCFL5-Tx mice. No significant differences were observed. (n = 7), t-test p < 0.05 (*), p < 0.01 (**), p<0.001 (***). **E)** mRNA expression by qPCR of *Dmrt*1*, Sox2* and *Dalz* in TCFL5^+/+^ and TCFL5^-/-^ mice. TCFL5 null mice present higher expression of *Dmrt*1*, Sox2* and *Dalz* than TCFL5 wild type mice. (n = 8), t-test p<0.05 (*), p<0.01 (**). **F)** *In silico* putative TCFL5 binding site in *Dazl*, *Dmrt1*, *Stag3*, *Syce1*, *Dcaf17*, *Spata16* promoters**. G-H)** CHIP assay to identify TCFL5 binding to *Stag3*, *Syce1* and *Dmrt1* promoters determined by qPCR (G) and PCR (F).


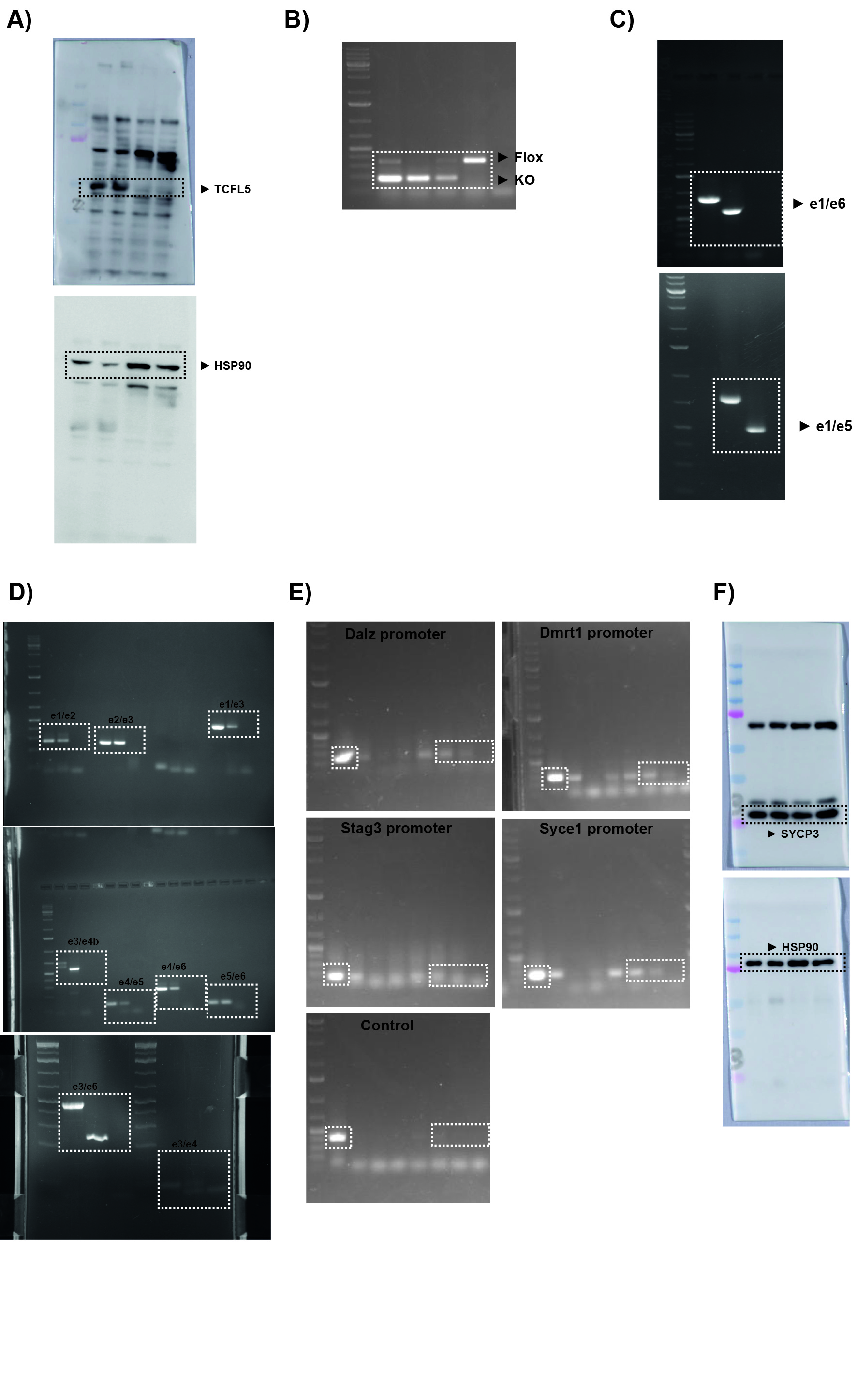


**Supplementary Figure 8. Complete membranes of Western-blot and PCR.** Complete membranes of WB corresponding to A) Figure 2B, B) Supplementary Figure 2C, C) Supplementary Figure 1C, D) Supplementary Figure 1D, E) Supplementary Figure 7G, F) Supplementary Figure 4.

| **Gene** | **Forward (5’-3’)** | **Reverse (5’-3’)** |
| --- | --- | --- |
| *e1/e2* | GACCGCTTCAACAGTATGCC | GTTACTAGAACTCTGCGTCTGTG |
| *e2/e3* | ACTCATGCTCTATACTGGAAGCTG | CTTCTCCCACTTTAATCCAAACCT |
| *e1/e3* | GACCGCTTCAACAGTATGCC | CTTCTCCCACTTTAATCCAAACCT |
| *e3/e4* | GCAGGTTTGGATTAAAGTGGGA | AAGAGCCTTCACCTACATTCTG |
| *e3/e4b* | GCAGGTTTGGATTAAAGTGGGA | ACAGCCAGGGCTACACATCT |
| *e3/e6* | GCAGGTTTGGATTAAAGTGGGA | TCAGTCCAGTCACTTGATCTCC |
| *e4/e5* | CGGAGCAGGTTTGGATTAAA | GGGTTGTTGCTTTATCTGTCTC |
| *e4/e6* | CGGAGCAGGTTTGGATTAAA | TCAGTCCAGTCACTTGATCTCC |
| *e5/e6* | AGTACATTCAGGAAAGACATGGG | TCAGTCCAGTCACTTGATCTCC |
| *e1/e6* | GACCGCTTCAACAGTATGCC | TCAGTCCAGTCACTTGATCTCC |
| *e1/e5* | GACCGCTTCAACAGTATGCC | GGGTTGTTGCTTTATCTGTCTC |
| *Aif* | TCCAGAGGCCGAAACAGAG | CATTTTGCCCCCTGATGAACC |
| *Bax* | TGAAGACAGGGGCCTTTTTG | AATTCGCCGGAGACACTCG |
| *Bcl2* | ATGCCTTTGTGGAACTATATGGC | GGTATGCACCCAGAGTGATGC |
| *Hif1a* | ACCTTCATCGGAAACTCCAAAG | ACTGTTAGGCTCAGGTGAACT |
| *18S* | GCAATTATTCCCCATGAACG | GGGACTTAATCAACGCAAGC |
| *Morc2a* | TGGCATCCTTGATCCTTTTC | AAGTACGCATGGGTGGAGAC |
| *Stag3* | TCCTCAGGCAGTGAGTCTTC | GTTCCCTGTGAGTCTCTGTCAT |
| *Syce1* | CTGGCCAGGAGAAGGAAAGTG | GTTGGCCTTCTCTTTGTCG |
| *Dalz* | TCGAAGGGCTATGGATTTGT | ACGTGGCTGCACATGATAAG |
| *Sox2* | TCGAGATAAACATGGCAATCA | AACCCAGCAAGAACCCTTTC |
| *Dmrt1* | CAGAGGACGATGGTCATC | TGTAGTAGGCGGGGTCTGATA |
| *Dcaf17* | TGACAGCTACTACGGAAGCAA | AATCACCGTTACAGCTTCTGG |
| *Dazl* promoter | CCACTTCTCTGTGCTACACC | TCTCTTTCCACCACCGCCTC |
| *Dmrt1* promoter | CTGGAGCCAGGTGGTAGAAATC | GCCAATGGTTGCTTGGAGGAG |
| *Stag3* promoter | GGACTGAAGGCAGCAGCAAAC | GCTAACCACGTTCCTGCCTC |
| *Syce1* promoter | GGCCATACTGCTCCACTTCC | GGCCTTTATGCTTGTTCATGC |
| Control promoter | CCAGCAATGAGCCAGCACAC | CCGTTAGATGTTGCATGTTCG |

**Supplementary Table 1.** Oligonucleotide sequences.

|  | **NCBI** | **Ensembl** | **Vega** | **Uniprot** |
| --- | --- | --- | --- | --- |
| Gene ID | 277353 | ENSMUST00000131881 | OTTMUSG00000016345 | - |
| *Tcfl5_e6* | NM_178254.3  BC108399.1 | ENSMUST0000037877 | OTTMUST00000039295 | Q32NY8 |
| *Tcfl5_e6∆5* | XM_006500653.1 | - | - | - |
| *Tcfl5_e6b* | AK132721.1 | ENSMUST00000131881 | - | Q3V133 |
| *Tcfl5_e4l* | AY234363.1  XM_006500652.1 | - | - | Q810E9 |
| *Tcfl5_e5* |  | ENSMUST00000161425 | OTTMUST0000086954 | ESQ5K1 |

**Supplementary Table 2.** Mice Tcfl5 sequences deposited in NCBI, Ensembl, Vega and Uniprot databases.

**Supplementary Table 3.** Top 100 most *Tcfl5*-correlated genes obtained from ARCHS4 web resource and Functional categories and Gene ontology analysis.
